# Perinatal suicidality: Risk factors in South African women with mental illness

**DOI:** 10.4102/sajpsychiatry.v26i0.1412

**Published:** 2020-08-24

**Authors:** Elsa du Toit, Dana Niehaus, Esme Jordaan, Liezl Koen, Roxane Jones, Jukka Leppanen

**Affiliations:** 1 Maternal Mental Health Clinic, Stikland Hospital, Cape Town, South Africa; 2 Department of Psychiatry, Faculty of Medicine and Health Sciences, Stellenbosch University, Cape Town, South Africa; 3 Panorama Healthcare Psychiatry, Panorama Medical Centre, Cape Town, South Africa; 4 Biostatistics Unit, Medical Research Council, Cape Town, South Africa, South Africa; 5 Department of Statistics and Population Studies, University of the Western Cape, Cape Town, South Africa; 6 Tampere Center for Child Health Research, University of Tampere School of Medicine, Tampere, Finland

**Keywords:** maternal mental health, perinatal psychiatry, suicidality, maternal mortality, unplanned pregnancy

## Abstract

**Background:**

Maternal Mortality is a global health concern. The lack of suicide data, particularly in low and middle income countries, is concerning and needs to be addressed.

**Aim:**

This study assessed suicidality and associated factors during pregnancy and the postpartum period amongst women with known psychiatric diagnoses.

**Setting:**

The study sample included pregnant South African women over the age of 18 years with a psychiatric disorder who presented at two maternal mental health clinics.

**Method:**

Suicidality was assessed by means of psychiatric interviews – the Mini International Neuropsychiatric Interview and the Montgomery Asberg Depression Rating Scale.

**Results:**

The results revealed that women were at a higher risk of experiencing suicidality if they had attempted suicide before, presented at a later gestation for psychiatric care or were employed. It was also clear that multiple assessments, carried out by means of clinical interviews and various scales, were necessary to screen suicidality successfully in pregnant women diagnosed with psychiatric illness.

**Conclusion:**

The results confirmed the view of the World Health Organization that in order to promote mental health and well-being, women’s health should be viewed contextually, not in isolation. Screening for and treatment of perinatal mental illness, including suicidality, are essential if we hope to meet the maternal morbidity and mortality targets of the United Nations by 2030.

## Introduction

Globally, suicide is the fourth leading cause of death of reproductive women, causing 8% – 9% of deaths in this age group.^1^ Suicide is also believed to be an important cause of maternal death during the perinatal period (i.e. conception to 12 months postpartum).^2,3,4,5^ The prevalence of maternal deaths varies highly between developed and developing countries (e.g. South Africa 134/100 000 [Savings Mothers 2017],^6^ or Papua New Guinea 870/100 000 vs. Australia 4/100 000).^7^ Maternal mortality remains a public health priority worldwide, and death rates because of medical illness such as pre-eclampsia are generally well documented.

In contrast, the lack of inclusive data on suicide deaths during pregnancy is alarming.^8^ This is particularly true for lower- and middle-income countries (LMICs) where the number of pregnancy-related deaths attributable to suicide remains largely unknown in spite of the World Health Organization’s (WHO) estimation that three-quarters of suicides occur in LMIC.^9^ Various factors, such as adverse economic and socio-demographic factors,^10,11^ discrimination against women and inappropriate and unresponsive healthcare services, are major risk factors for women’s health in LMIC.^7^ A systemic review performed by Fuhr et al.^12^ found that suicide deaths account for only 1% – 2% of pregnancy-related deaths in LMICs. However, stigma, misreporting and inaccurate recording of cause of death contribute to inaccuracy of data, and the true proportion of deaths attributable to suicide in pregnant women living in LMICs may not be represented in available data. In fact, a recent study from Sri Lanka indicated that if suicide deaths are properly classified and reported, it may account for 18% of pregnancy-related mortality.^13^ In South Africa, the current maternal suicide rate of 1% should be interpreted with caution because three-quarters of non-natural causes of death are not adequately classified.^14^

Although the risk of death from suicide appears to be higher in non-pregnant women than pregnant women, a high prevalence of suicidality is found amongst pregnant women.^15,16,17^ Suicidality refers to a spectrum that ranges from suicidal ideation (suicide thoughts and plans) to intentional self-harm, suicide attempts and actual suicide.^18^ In South African women, suicidality rates are between 7% and 28% (Saving Mothers Report 2017). The perinatal period offers a unique opportunity to detect and manage suicidality by means of the regular contacts pregnant women have with healthcare services.

In a recent article, Onah et al.^19^ highlighted the importance of paying attention to contextual factors that may contribute to suicidality in the perinatal period, especially in LMICs. The majority of studies reported a positive association between suicidality and poverty.^10,11,20^ Apart from the commonly cited barriers to treatment in poorly resourced settings, such as a lack of transport and long waiting periods in public sector care facilities,^21^ domestic violence,^22^ unplanned pregnancy^23,24,25^ and unemployment^26,27,28,29,30^ are all factors that increase the risk for perinatal mental illness, and alongside this, suicidality.

The presence of mental health disorders is the strongest risk factor for suicidality in the general population,^31,32^ pregnant women^33^ and women in the postpartum period.^34^ Women diagnosed with mental disorders such as schizophrenia or bipolar disorder are more likely to commit suicide during pregnancy.^35^ However, future suicide attempts are not the only concern. Suicidality during the perinatal period is associated with the risk of postpartum depression,^36,37,38^ and an increase in all-cause mortality rates.^39,40^

Although much has been written about maternal mental illness in the perinatal period, little is known about perinatal suicidality and its risk factors.^13,41^ In this naturalistic descriptive study, we examined suicidality in women diagnosed with a psychiatric illness over the course of pregnancy and the first 12 weeks postpartum, with the aim of identifying factors linked to an increased risk for suicidality. Given the study setting (tertiary perinatal psychiatric clinics) and the known links between mental illness and suicidality, on the one hand, and the perinatal period and suicidality, on the other, it follows that the group of women who constituted the study sample was at a particular risk for suicidality. Identifying additional risk factors – including both contextual factors and those linked to psychiatric diagnoses – may prove valuable in the development of suicide screening and prevention programmes, especially in LMICs like South Africa where the prevalence of perinatal depression and suicide is high.^15^

## Methodology

This quantitative, descriptive study took place between April 2011 and July 2017 at two maternal mental health clinics in the Cape Town metropole: The Maternal Mental Health Outpatient Clinic at the state-owned Stikland Hospital and the private-owned Panorama Healthcare Psychiatry Centre.

The authors aimed to assess suicidality in women 18 years and older with a DSM-IV-TR axis-1 diagnosis of psychiatric illness. Women, who provided written informed consent, were studied from first presentation at a recruitment site until 12 weeks postpartum. The study was approved by the research ethics committee of Stellenbosch University (reference number: N12/10/071), and permission was obtained from Stikland Psychiatric Hospital and the Provincial Government of the Western Cape.

As women presented for their initial interview at different gestational stages, study entry was possible in any of the three trimesters of pregnancy. A psychiatric assessment by either a qualified psychiatrist, with a minimum of 5 years’ experience, or a psychiatric registrar under the supervision of such a psychiatrist was completed for each participant. The information was extracted onto a data capturing platform by a dedicated data capturer using a pre-selected coding system.

The interviewer enquired about past and current suicidality at every assessment, as per standard clinical assessment interview. In addition to this, the clinician made use of two research scales, the Mini International Neuropsychiatric Interview (MINI) and the Montgomery Asberg Depression Rating Scale (MADRS) to further assess suicidality. Both scales were validated, widely available with sensitivity and specificity comparable to other scales used in clinical settings.^42,43,44^ Section C of the MINI comprised nine questions that explore past and present suicidal thoughts and behaviours. The MINI enquired about thoughts of death, suicide and/or self-harm. It further explored the presence of a suicide plan, preparation to execute such a plan and whether any self-harming behaviour or actual suicide attempts was evident in the past month. Item 9 of the MINI asked about lifetime suicide attempts, and therefore this question was excluded during analysis. The MADRS enquired about current (past week) suicidal thoughts by means of a single question about suicide thoughts or the feeling that life is not worth living. The participant can score between 0 (enjoys life) and 6 (explicit plans for suicide).

Suicidality was considered present in any of the following cases:

- suicide attempt was reported or ideation or plan was expressed during a clinical interview at any of the visits to the clinic
- positive indication of suicidal ideation or attempt on the MINI interview (excluding item 9)(3) suicide item on MADRS scored 2 (fleeting suicidal thoughts, weary of life) or more at any visit.

The number of positive suicidal responses based on the above criteria and the number of weeks in the study were counted for each woman, and the incidence rate was expressed as the number of total positive suicidal responses divided by the total weeks in the study of all women, expressed per 100 weeks. Multiple iterations were possible to reach the final suicidal score: Individual A could, for example, have responded positive on the MINI items at visit 1, thus scoring one point, and indicated suicidal ideation on a MADRS assessment at visit 3, scoring another point, ending up with a total of two points. Individual B could have responded positive on questions regarding suicidal ideation or plan during the clinical interview at visit 2 and could also have scored positive on the MADRS item 10 at the same visit, leading to a final score of two. This approach was followed to allow the clinician to gain a better understanding of the number of red flags (positive suicidal responses) shown by a patient across the study period. This was very valuable, as it was not uncommon to find patients endorsing the MADRS item at a specific visit, but not the clinical questions relating to suicidal ideation at the same visit. A Poisson-regression model with a log link and the total number of weeks as offset was used to assess the risk factors for suicidality. It was possible for a woman to enter the study more than once with subsequent pregnancies. A ‘participant’ is defined as a woman with a single or multiple pregnancies at entry to the study. Correlations as a result of the same women having multiple pregnancies during the study were accounted for by an exchangeable correlation structure in the Generalized estimating equation (GEE) model. As the data were obtained from two clinics (strata), these were included as a fixed effect in all regression models.

As this study aimed to reflect findings from a ‘real-world’ setting, it did neither interfere with nor dictate care as usual practice. If women were found to screen positive for suicidality, the clinician would continue with standard care practice as dictated by the clinical scenario; for example, the participant might be admitted to hospital or referred to social services. On the other hand, if a woman chose not to participate in the study, her care would not be compromised in any way.

### Ethical consideration

This study was approved by the Health Research Ethics Committee of Stellenbosch University (project reference number: 4286; Ethics reference number: N12/10/071). All procedures performed in this study were in accordance with the ethical standards of the institutional research committee and complied with the 1964 Helsinki declaration and its later amendments or comparable ethical standards.

## Results

Of the 263 individual women enrolled in the study, 243 had one pregnancy with one baby, 15 had two pregnancies with one baby at each pregnancy, one had three pregnancies with one baby at each pregnancy and four had one pregnancy with twins. In total, there were 280 pregnancies (called participants from here on), resulting in 284 babies. As part of the care-as-usual protocol at the Maternal Mental Health Service, all 280 participants underwent a semi-structured clinical interview. In addition, 241 participants completed the MINI section C, and 169 participants were evaluated by administration of the MADRS. The demographic information of the 280 participants is summarised in Table 1. The mothers were mostly of mixed ancestry (46%) or Caucasian (43%), with a mean age of 31 years. Most women had a Grade 11 or higher education (76%), but less than half were employed, and 48% were part of a low-income household.

**TABLE 1:** The number of women, number of suicides and incidence of suicidality during pregnancy (incidence rate and 95% confidence interval per 100 women weeks in the study) by maternal, demographic and pregnancy characteristics.

Demographic characteristic	Total number of women		Number of women with suicidality	Incidences of suicidality	Woman weeks in the study	IR	95% CI	*p*	IRR	95% CI
	*n*	%								
Overall	280	-	71	101	5446	1.8	1.4–2.3	-	-	-
**Age (years)**										
18–34	197	70.4	46	67	3799	1.6	1.1–2.2	0.149	1.4	0.9–2.4
35–46	83	29.6	25	34	1647	2.3	1.5–3.4	-	-	-
**Education level**										
Lower than Grade 11	65	23.2	25	30	1094	2.2	1.2–4.0	0.473	1.5	0.5–4.3
Secondary school: Grade 11–12	100	35.7	24	35	1570	2.0	1.1–3.5	0.597	1.3	0.4–3.5
Tertiary education	114	40.7	22	36	2778	1.5	0.8–2.8	-	-	-
Missing	1	0.4	-	-	-	-	-	-	-	-
**Employment status**										
Employed	137	48.9	24	37	3125	2.5	1.7–3.5	0.029	1.9	1.1–3.5
Unemployed	142	50.7	46	63	2291	1.3	0.8–2.0	-	-	-
Missing	1	0.4	0	0	0	-	-	-	-	-
**Relationship status**										
Married	164	48.9	39	56	3656	1.7	1.2–2.4	0.677	1.1	0.6–2.2
Unmarried	116	41.4	32	45	1790	2.0	1.2–3.2	-	-	-
**Income level†**										
> R4 166/month (+$292)	151	53.9	33	51	3225	2.2	1.4–3.5	0.193	1.7	0.8–3.9
**<** R4 166/month (+$292)	129	46.1	38	50	2221	1.3	0.8–2.2	-	-	-
**Previous suicide attempts**										
No	184	65.7	31	42	3910	1.2	0.8–1.7	0.007	1.0	1.3–4.1
Yes	96	34.3	40	59	1981	2.6	1.8–3.8	-	2.3	-
**Gestation at presentation**										
1–13	127	45.3	29	39	3249	1.2	0.8–1.8	0.249	1.4	3.1–9.9
14–26	103	36.8	21	33	1807	1.7	1.1–2.7	0.0001	5.6	3.1–9.9
27–40	49	17.5	21	29	389	6.8	4.3–10.7	-	-	-
Missing	1	0.4	0	-	-	-	-	-	-	-
**Weeks at delivery**										
8–36 weeks	41	14.6	10	15	718	2.1	1.2–3.7	0.569	1.0	-
37 weeks	32	11.4	9	12	739	1.6	0.8–3.2	-	-	-
38 weeks	54	19.3	14	20	1299	1.6	0.9–2.7	-	-	-
39 weeks	50	17.9	12	14	1270	1.1	0.7–1.9	-	-	-
40–42 weeks	46	16.4	11	18	1086	1.5	0.7–2.7	-	-	-
Missing data	57	20.4	-	-	-	-	-	-	-	-
**Gravida**										
1	75	36.0	12	19	1398	1.3	0.7–2.5	0.540	1.4	0.6–3.0
2	82	29.3	18	27	1597	1.8	1.1–2.9	-	1.5	0.7–3.4
3	62	22.1	17	26	1298	2.0	1.2–3.2	-	-	-
4 or more	61	21.8	24	29	1153	2.2	1.5–3.2	-	1.6	0.8–3.5
**Pregnancy planning**										
Planned and wanted	150	53.6	33	51	3391	1.7	1.2–2.4	0.947	1.0	0.5–1.8
Discordant (unsure)	95	33.9	24	32	1554	1.7	1.0–2.7	0.131	1.7	0.8–3.5
Unplanned and unwanted	34	12.1	14	18	497	2.9	1.6–5.3	-	-	-
Missing	1	0.4	-	-	-	-	-	-	-	-
**Interactions**										
Wanted pregnancy and employed	129	46.1	23	36	3325	1.2	0.8–1.9	-	-	
Wanted pregnancy and unemployed (*n* = 108) or Unwanted pregnancy and employed (*n* = 8)	116	41.4	30	40	2145	1.7	1.1–2.5	-	1.4	0.7–2.7
Unwanted pregnancy and unemployed	33	11.8	17	24	387	5.3	2.9–9.5	0.01	4.4	2.0–9.8

CI, confidence interval; IR, incidence rate; IRR, incidence rate ratio.

† , Income level divided according to government cut-off.

More than half of the women (54%) only presented to the Maternal Mental Health clinics after their first trimester (Table 1). Just over half of the pregnancies (54%) were planned and wanted, whilst the rest were unplanned and/or unwanted. Tobacco smoking (38%) and alcohol use (19%) were the main substances used, followed by methamphetamine (8%) and cannabis (6%). Most women had a single psychiatric diagnosis (64%) and were on a single psychotropic medication (64%) (Table 2). The most common psychiatric diagnosis (multiple diagnoses were possible) were major depressive disorder (MDD) (*n* = 164, 59%), bipolar spectrum disorder (BSD, including bipolar disorder I, II and bipolar disorder not otherwise specified) (*n* = 61, 22%), borderline personality and traits (BPT) (*n* = 54, 19%), schizophrenia spectrum disorders (*n* = 47, 17%), generalised anxiety disorder (GAD) (*n* = 44, 16%), substance use disorders (*n* = 34, 12%), panic disorder (*n* = 12, 4%), obsessive compulsive disorder (OCD) (*n* = 11, 4%) and post-traumatic stress disorder (PTSD) (*n* = 6, 2%).

**TABLE 2:** The number of women, number of suicides and incidence of suicidality during pregnancy (incidence rate and 95% confidence interval per 100 women weeks in the study) by psychiatric diagnosis and treatment.

Demographic characteristic	Total number of women		Number of women with suicidality	Incidences of suicidality	Woman weeks in study	IR	95% CI	*p*	IRR	95% CI
	*n*	%								
**Diagnosis**										
Single diagnosis	180	64.3	45	66	3256	1.9	1.3–2.7	-	-	-
Two or more diagnoses	100	35.7	26	35	2190	1.6	1.1–2.3	0.521	0.9	0.5–1.4
**Specific diagnosis present versus absent** ‡										
MDD present† versus absent	164	58.6†	46 versus 25	64 versus 37	3196 versus 2250	2.3 versus 1.3	1.7–3.1 versus 2–7.2	0.036	1.9	1.1–3.4
BSD present† versus absent	61	21.8†	14 versus 0	23 versus 0	1362 versus 0	0.8 versus 1.4	0.8–2.5 versus 1.4–2.6	0.308	0.7	0.4–1.4
BPT present† versus absent	54	19.3†	23 versus 0	37 versus 0	1145 versus 0	2.8 versus 1.5	1.9–4.2 versus 1.1–2.1	0.026	1.9	1.2–3.1
Schizophrenia Spectrum present† versus absent	47	16.8†	10 versus 61	11 versus 90	809 versus 4637	0.9 versus 2.0	0.4–1.8 versus 1.5–2.6	0.023	0.5	0.2–0.9
GAD present† versus absent	44	15.7†	4 versus 67	6 versus 95	1068 versus 4378	0.7 versus 2.1	0.2–1.9 versus 1.6–2.7	0.020	0.3	0.1–1.0
Substance use disorder present† versus absent	34	12.1	11 versus 60	17 versus 84	701 versus 4745	1.9 versus 1.8	1.1–3.3 versus 1.3–2.4	0.863	1.1	0.6–1.9
**Medication use**										
Single medication (including treatment-naïve patients – *n* = 5, 14.5%)	181	64.6	43	56	3279	1.8	1.3–2.4	-	-	-
Polypharmacy	99	35.4	28	45	2167	1.8	1.2–2.8	0.926	1.0	0.6–1.7
**Substance use during pregnancy**										
Alcohol use										
No	224	80.0	51	75	4459	1.7	1.2–2.3	-	-	-
Yes	54	19.3	20	26	982	2.3	1.5–3.6	0.241	1.4	0.8–2.3
Missing	2	0.7	-	-	-	-	-	-	-	-
Tobacco smoking										
No	173	60.8	37	50	3629	1.5	1.0–2.1	-	-	-
Yes	107	38.2	34	51	1817	2.4	1.6–3.6	0.058	1.7	1.0–2.8
Missing	0	0.0	-	-	-	-	-	-	-	-
Other substances use										
No	240	85.7	59	81	4799	1.7	1.3–2.2	-	-	-
Yes (cannabis use = 5.7%, methamphetamine use = 7.9%, other substances§ = 2.5%)	36	12.9	11	19	620	2.4	1.3–4.5	0.323	1.4	0.7–2.7

BSD, bipolar spectrum disorders; BPT, borderline personality and traits; CI, confidence interval; GAD, generalised anxiety disorder; IR, Incidence rate; IRR, Incidence rate ratio; MDD, major depressive disorder.

† , Incidence rate.

‡ , multiple diagnosis possible.

§ , Other substances = codeine, mandrax, benzodiazepine and benzodiazepine receptor agonists.

### Suicide attempts

The clinical interview revealed that just under a third (27.5%, 95% confidence interval [CI]: 21.9–34.6) of participants had attempted suicide during their lifetime, prior to study inclusion. Seven women attempted suicide during enrolment in the study. Forty-five women who had a history of suicide attempts, had more than one previous attempt. The majority of suicide attempts were by means of a medication overdose alone (*n* = 57) or in combination with an overdose (*n* = 5).

### Suicidality during study participation

Overall, 71 (24.3%, 95% CI: 19.5–30.3) women reported suicidal ideation, plan or attempt according to the criteria. Eighteen women reported current suicidal ideation or plan during a clinical interview (three women reported suicidal ideation at two visits resulting in a total of 21 reports of suicidal ideation). In the MINI evaluation, a total of 50 suicidality responses were counted, with one-fifth of the participants reporting a wish to be dead (item 2) and 32 admitting to suicidal ideation (item 4). A total of 23 participants met the cut-off anchor score for question 10 of the MADRS, thus confirming suicidal ideation.

In total, 101 suicidality responses were counted for the 71 women during the study. Counting 5446 total women weeks in the study, this results in a suicidality incidence rate (IR) of 1.8 (95% CI: 1.4–2.3) per 100 women weeks in the study (Table 1).

Results revealed that women were at a significantly higher risk of experiencing suicidality during their pregnancy if they had attempted suicide before (*p* = 0.007) the current pregnancy. The IR for the group who had never attempted suicide was 1.3 (95% CI: 0.8–1.9) versus 2.7 (95% CI: 1.9–3.9) for the attempted suicide group – more than twice the incidence rate in the exposure group compared to the group not reporting previous suicide attempts (IR ratio = 2.2; 95% CI: 1.2–3.8).

Compared to early presentation (≤ 13 weeks), late presentation (> 27 weeks) to mental health clinics was a highly significant risk (*p* = 0.0001) for suicidality in women. The IR for the group who presented in the first trimester was 1.2 (95% CI: 0.8–1.8) versus 6.8 (95% CI: 4.3–10.7) for women who presented after the end of the second trimester (> 27 weeks) (Table 1).

Women who were employed were significantly more likely to experience suicidality (IR = 2.5; 95% CI: 1.7–3.5) than those who were unemployed (IR = 1.3; 95% CI: 0.8–2.0) (*p* = 0.029) (Table 1). However, when interactions amongst employment and wanted pregnancy variables were considered, women who were unemployed and had unwanted pregnancies had a significantly higher risk of suicidality (IR = 5.4; 95% CI: 3.0–9.8), compared to women who were employed and had wanted pregnancies (IR = 1.3; 95% CI: 0.8–2.1).

This study confirmed that the presence of MDD (*p* = 0.036) and BPT (*p* = 0.026) are positively associated with suicidality, whilst schizophrenia spectrum disorders (*p* = 0.023) and GAD (*p* = 0.020) were negatively associated with a heightened risk of suicidality. Although women with BSD displayed a higher IR of suicidality than those without (1.4 vs. 0.8), it was not statistically significant (*p* = 0.308).

In a multiple regression model, the independent risk factors for suicidality were presentation for pregnancy at 27 weeks or later (incidence rate ratio [IRR] = 2.6; 95% CI: 1.5–4.4, *p* = 0.0009) and prior suicide attempts (IRR [third vs. first trimester] = 6.4; 95% CI: 3.6–11.4, *p* = 0.001).

## Discussion and conclusion

Maternal suicidality, a risk factor for maternal suicide, occurred at high rates amongst women diagnosed with a psychiatric disorder. The IR of 1.8 per 100 women weeks implies that there was one suicide behaviour in every 55 weeks of pregnancy.

This study confirmed that suicidality during the perinatal period warranted further investigation, not only because it provides an opportunity to prevent future suicide attempts but also because suicidal ideation is associated with other factors that further compromise maternal and infant health and well-being, such as the presence of a mental illness,^24,45^ tobacco^46,47^ and alcohol use,^46,48^ and non-suicide-related mortality.^39,40^

The prevalence of suicidality found amongst our study population was 24%. These rates echoed the upper end of percentages reported by the Saving Mothers Report (28%) but is substantially higher than other previously reported rates for the perinatal period (i.e. 14%,^16^ 2.7%^24^ and 2.6%^49^). This may be explained by the fact that all the women in the current study had previously diagnosed psychiatric disorders. Psychiatric disorders have consistently been described as a risk factor for suicide attempts.^9,50,51,52^ In fact, Newport et al.^53^ found similarly high rates of suicidality (between 16.7% and 27.8%, depending on which rating-scale is used) amongst women diagnosed with mental illness during the perinatal period. More than a third (35%) of substance-dependent pregnant women studied by^34^ also reported current suicidal ideation.

Lindahl et al.^16^ cautioned against the use of a single item to assess suicidality. A major strength of our study was the application of multiple measures (i.e. patient report, semi-structured interview, MINI and MADRS) to assess suicidality. Further strength is that the assessments were carried out at different time points during the perinatal period.

Previous suicide attempts, employment and late presentation (> 27 weeks gestation) at maternal mental healthcare services were identified as factors that further increased the risk of suicidality in women with mental illness during the perinatal period. A previous study^25^ found an association between suicidality and pregnancy planning. Pregnancy planning and attitude towards the pregnancy (wanted and unwanted) initially did not appear to impact significantly on suicidality in this study (*p* = 0.131). However, when interactions between employment status and pregnancy status (wanted and unwanted) were considered, women who experienced unwanted pregnancies in combination with unemployment had a significantly elevated risk of suicidality (*p* = 0.0005) – a finding that makes a strong case for the importance of viewing maternal mental health contextually.

The well-known associations between mental illness, previous suicide attempts and future suicide attempts^24,53,54,55^ were confirmed by this study, but results revealed that the risk is not equal for all Diagnostic and Statistical Manual of Mental Disorders-Fourth Edition (Text Revision) (DSM-IV-TR) diagnoses. The elevated risk of suicidality amongst women with BPT supports the findings of a previous study.^56^ The fact that BSD was not associated with a heightened suicide risk is interesting, but the different diagnoses on the bipolar spectrum should be studied separately with larger numbers of perinatal women before any conclusions can be drawn. This study indicates that preventive measures should target women with mental illness, especially those with a history of suicide attempts and diagnoses of MDD and/or BPT. Incorporating screening for mental illness and suicidality in routine antenatal assessments may be an appropriate place to start.

The finding of employment as risk factor for suicidality is interesting. Gender stereotypes prescribing women to ‘put family first’ is still prevalent in many cultures,^57^ and women may struggle to balance the demands of a career and a family.^58,59^ Juggling a career with motherhood may prove challenging, and the associated restructuring of professional identity may lead to negative feelings.^60^ On the other hand, the very high IR of suicidality amongst unemployed women with unwanted pregnancies may have been influenced by the cumulative effect of unwelcome motherhood and lack of income. The finding of increased suicidality in women with these seemingly opposite sets of circumstances, once again, shows the importance of a holistic approach to maternal mental health, which takes into account both socio-economic and cultural barriers.

The third risk factor for suicidality during the perinatal period for women with mental illness identified by this study is late presentation to maternal mental health services. It is well-known that perinatal mental illness is under-recognised and under-treated, leaving many women vulnerable to the consequences of untreated mental illness, including suicidal ideation and suicide.^61^ It is a tragedy that suicide is still considered one of the leading causes of maternal deaths.^2,3,4,5^ If we want to meet the third goal of the United Nations (UN) 2030 Agenda for Sustainable Development, namely, improving maternal health and well-being,^62^ screening for and treating mental illness during the perinatal period as a part of standard antenatal care should be mandatory – as women are particularly vulnerable during this period, they must have frequent contact with healthcare services, providing the ideal opportunity for addressing mental health concerns and suicidality.

## References

[1] Lozano R, Naghavi M, Foreman K, et al. Global and regional mortality from 235 causes of death for 20 age groups in 1990 and 2010: A systematic analysis for the Global Burden of Disease Study 2010. Lancet. 2012;380(9859):2095–2128. 10.1016/S0140-6736(12)61728-0 23245604 PMC10790329

[2] Cantwell R, Clutton-Brock T, Cooper G, et al. Saving mothers’ lives: Reviewing maternal deaths to make motherhood safer: 2006–2008. The eighth report of the confidential enquiries into maternal deaths in the United Kingdom. BJOG. 2011;118(1):1–203. 10.1111/j.1471-0528.2010.02847.x 21356004

[3] Oates M. Suicide: The leading cause of maternal death. B J Psychiatry. 2003;183(4):279–281. 10.1192/bjp.183.4.279 14519602

[4] Shadigian EM, Bauer ST. Pregnancy-associated death: A qualitative systematic review of homicide and suicide. Obstet Gynecol Surv. 2005;60(3):183–190. 16570396 10.1097/01.ogx.0000155967.72418.6b

[5] Turecki G, Brent DA. Suicide and suicidal behaviour. Lancet. 2016;387(10024):1227–1239. 10.1016/S0140-6736(15)00234-2 26385066 PMC5319859

[6] Department of Health South Africa. Savings Mothers 2017: Annual Report on Confidential inquiries into maternal death in South Africa. Department of Health South Africa; 2017.

[7] Hinton R, Earnest J. The right to health: Overcoming inequalities and barriers to women’s health in Papua New Guinea. Women’s Stud Int Forum. 2010;33(3):180–187. 10.1016/j.wsif.2009.12.006

[8] Mangla K, Hoffman C, Trumpff C, O’Grady S, Monk C. Maternal self-harm deaths: An unrecognized and preventable outcome. AJOG. 2019;221(4):295–303. 10.1016/j.ajog.2019.02.056 30849358

[9] World Health Organization (WHO). Preventing suicide: A global imperative [homepage on the Internet]. 2014 [cited 2019 May 26]. Available from: https://www.who.int/mental_health/suicide-prevention/world_report_2014/en/

[10] Dewing S, Tomlinson M, Le Roux I, Chopra M, Tsai AC. Food insecurity and its association with co-occurring postnatal depression, hazardous drinking, and suicidality among women in peri-urban South Africa. J Affect Disord. 2013;150(2):460–465. 10.1016/j.jad.2013.04.040 23707034 PMC3762324

[11] Rochat TJ, Bland RM, Tomlinson M, Stein A. Suicide ideation, depression and HIV among pregnant women in rural South Africa. Health. 2013;5(3A):650–661. 10.4236/health.2013.53A086

[12] Fuhr DC, Calvert C, Ronsmans C, et al. Contribution of suicide and injuries to pregnancy-related mortality in low-income and middle-income countries: A systematic review and meta-analysis. Lancet Psychiatry. 2014;1(3):213–225. 10.1016/S2215-0366(14)70282-2 26360733 PMC4567698

[13] Agampodi S, Wickramage K, Agampodi T, et al. Maternal mortality revisited: The application of the new ICD-MM classification system in reference to maternal deaths in Sri Lanka. Reprod Health. 2014;11:17. 10.1186/1742-4755-11-17 24571652 PMC3937524

[14] Statistics South Africa. Mortality and causes of death in South Africa: Findings from death notification [homepage on the Internet]. 2015 [cited 2019 May 26]. Available from http://www.statssa.gov.za/?page_id=1854&PPN=P0309.3&SCH=6987

[15] Gelaye B, Kajeepeta S, Williams MA. Suicidal ideation in pregnancy: An epidemiologic review. Arch Womens Ment Health. 2016;19(5):741–751. 10.1007/s00737-016-0646-0 27324912 PMC5023474

[16] Lindahl V, Pearson JL, Colpe L. Prevalence of suicidality during pregnancy and the postpartum period. Arch Womens Ment Health. 2005;8:77–87. 10.1007/s00737-005-0080-1 15883651

[17] Orsolini L, Valchera A, Vecchiotti R, et al. Suicide during perinatal period: Epidemiology, risk factors, and clinical correlates. Front Psychiatry. 2016;7:138. 10.3389/fpsyt.2016.00138. 27570512 PMC4981602

[18] Franklin JC, Ribeiro JD, Fox KR, et al. Risk factors for suicidal thoughts and behaviors: A meta-analysis of 50 years of research. Psychological Bulletin 2017;143(2):187–232. 27841450 10.1037/bul0000084

[19] Onah MN, Field S, Bantjes J, Honikman S. Perinatal suicidal ideation and behaviour: Psychiatry and adversity. Arch Womens Ment Health. 2017;20(2):321–331. 10.1007/s00737-016-0706-5 28032214 PMC5344952

[20] Dumith SC, Demenech LM, Carpen MX, Nomiyam S, Neiva-Silv L, De Mol CL Suicidal thought in southern Brazil: Who are the most susceptible? Journal of affective disorders. 2020;260:610–616. 10.1016/j.jad.2019.09.046 31541972

[21] Vasco M, Pandya S, Van Dyk D, et al. Maternal critical care in resource-limited settings. Narrative review. Int J Obstet Anesth. 2019;37:86–95. 10.1016/j.ijoa.2018.09.010 30482717

[22] Supraja TA, Thennarasu K, Satyanarayana VA, et al. Suicidality in early pregnancy among antepartum mothers in urban India. Arch Womens Ment Health. 2016;19:1101–1108. 10.1007/s00737-016-0660-2 27565804

[23] Abajobir AA, Maravilla JC, Alati R, Najman JM. A systematic review and meta-analysis of the association between unintended pregnancy and perinatal depression. J Affect Disord. 2016;192:56–63. 10.1016/j.jad.2015.12.008 26707348

[24] Gavin AR, Tabb KM, Melville JL, Guo Y, Katon W. Prevalence and correlates of suicidal ideation during pregnancy. Arch Womens Ment Health. 2011;14:239–246 10.1007/s00737-011-0207-5 21327844 PMC3724526

[25] Ishida K, Stupp P, Serbanescu F, Tullo E. Perinatal risk for common mental disorders and suicidal ideation among women in Paraguay. Int J Gynecol and Obstet. 2010;110(3):235–240. 10.1016/j.ijgo.2010.03.027 20472235

[26] Chang T, Chen W. Revisiting the relationship between suicide and unemployment: Evidence from linear and nonlinear cointegration. Econ Syst. 2017;41(2):266–278. 10.1016/j.ecosys.2016.06.004

[27] Chen J, Choi YJ, Mori K, Sawada Y, Sungano Y. Socio-economic studies on suicide: A survey. J Econ Surv. 2012;26(2):271–306. 10.1111/j.1467-6419.2010.00645.x

[28] Milner A, Hjelmeland H, Arensman EE, Leo D. Social-environmental factors and suicide mortality: A narrative review of over 200 articles. Sociol Mind. 2013;3(2):137–148. 10.4236/sm.2013.32021

[29] Milner A, Page A, LaMontagne AD. Cause and effect in suicides on unemployment, mental health and suicide: A meta-analytic and conceptual review. Psychol Med. 2014;44(5):909–917. 10.1017/S0033291713001621 23834819

[30] Milner A, Page A, LaMontagne AD. Long-term unemployment and suicide: A systematic review and meta-analysis. PLoS One. 2013;8(1):e51333. 10.1371/journal.pone.0051333 23341881 PMC3547020

[31] Nock MK, Hwang I, Sampson NA, Kessler RC. Mental disorders, comorbidity and suicidal behavior: Results from the National Comorbidity Survey Replication. Mol Psychiatry. 2010;15:868–876. 10.1038/mp.2009.29 19337207 PMC2889009

[32] Plener PL, Schumacher TS, Munz LM, Groschwitz RC. The longitudinal course of non-suicidal self-injury and deliberate self-harm: A systematic review of the literature. Borderline Personal Disord Emot Dysregul. 2015;2(2). 10.1186/s40479-014-0024-3 PMC4579518 26401305

[33] Bonari L, Pinto N, Ahn E, et al. Perinatal risks of untreated depression during pregnancy. Can J Psychiatry. 2004;49(11):726–735. 10.1177/070674370404901103 15633850

[34] Gold KJ, Singh V, Marcus SM, Palladino CL. Mental health, substance use and intimate partner problems among pregnant and postpartum suicide victims in the National Violent Death Reporting System. Gen Hosp Psychiatry. 2012;34(2):139–145. 10.1016/j.genhosppsych.2011.09.017. 22055329 PMC3275697

[35] Khalifeh H, Brauer R, Toulmin H, Howard LM. Perinatal mental health: What every neonatologist should know. Early Hum Dev. 2015;91(11):649–653. 10.1016/j.earlhumdev.2015.08.010 26386609

[36] Eggleston A, Calhoun P, Svikis D, et al. Suicidality, aggression, and other treatment considerations among pregnant, substance-dependent women with posttraumatic stress disorder. Compr Psychiatry. 2009;50(5):415–423. 10.1016/j.comppsych.2008.11.004 19683611 PMC3596016

[37] Gausia K, Fisher C, Ali M, Oosthuizen J. Antenatal depression and suicidal ideation among rural Bangladeshi women: A community-based study. Arch Womens Ment Health. 2009;12(5):351–358. 10.1007/s00737-009-0080-7. 19468825

[38] Paris R, Bolton R, Weinberg M. Postpartum depression, suicidality, and mother-infant interactions. Arch Womens Ment Health. 2009;12:309–321. 10.1007/s00737-009-0105-2 19728036

[39] Batterham PJ, Calear AL, Mackinnon AJ, Christensen H. The association between suicidal ideation and increased mortality from natural causes. J Affect Disord. 2013;150(3):855–860. 10.1016/j.jad.2013.03.018 23618327

[40] Cain DS, Loprinzi PD. Suicidal ideation and all-cause mortality risk. Best Pract Ment Health. 2018;14(2):1–8.

[41] Gressier F, Guillard V, Cazas O, et al. Risk factors for suicide attempt in pregnancy and the post-partum period in women with serious mental illnesses. J Psychiatr Res. 2017;84:284–291. 10.1016/j.jpsychires.2016.10.009 27810668

[42] Sheehan DV, Lecrubier Y, Sheehan KH, et al. The Mini-International Neuropsychiatric Interview (M.I.N.I.): The development and validation of a structured diagnostic psychiatric interview for DSM-IV and ICD-10. J Clin Psychiatry. 1998;59(20):22–33; quiz 34–57. 9881538

[43] Van Heyningen T, Myer L, Onah M, et al. Antenatal depression and adversity in urban South Africa. J Affect Disord. 2016;203:121–129. 10.1016/j.jad.2016.05.052 27285725 PMC5777271

[44] Wikberg C, Nejati S, Larsson MEH, et al. Comparison between the Montgomery-Asberg Depression Rating Scale–Self and the Beck Depression Inventory II in primary care. Prim Care Companion CNS Disord. 2015;17(3). 10.4088/PCC.14m01758 PMC4578910 26644958

[45] Asad N, Karmaliani R, Sullaiman N, et al. Prevalence of suicidal thoughts and attempts among pregnant Pakistani women. AOGS 2010;89(12):1545–1551. 10.3109/00016349.2010.526185 PMC3918941 21050149

[46] Czeizel AE. Attempted suicide and pregnancy. J Inj Violence Res. 2011;3(1):45–54. 10.5249/jivr.v3i1.77 21483214 PMC3134915

[47] Tavares D, Quevedo L, Jansen K, et al. Prevalence of suicide risk and comorbidities in postpartum women in Pelotas. Braz J Psychiatry. 2012;34(3):270–276. 10.1016/j.rbp.2011.12.001 23429772

[48] Farias DR, Pinto TdJP, Teofilo MMA, et al. Prevalence of psychiatric disorders in the first trimester of pregnancy and factors associated with current suicide risk. Psychiatry Res. 2013;210(3):962–968. 10.1016/j.psychres.2013.08.053 24090486

[49] Borges G, Angst J, Nock M, et al. A risk index for 12-month suicide attempts in the National Comorbidity Survey Replication (NCS-R). Psychol Med. 2006;36(12):1747–1757. 10.1017/S0033291706008786 16938149 PMC1924761

[50] Nock MK, Borges G, Bromet EJ, et al. Cross-national prevalence and risk factors for suicidal ideation, plans, and attempts. B J Psychiatry. 2008;192(2):98–105. 10.1192/bjp.bp.107.040113 PMC2259024 18245022

[51] O’Connor RC, Smyth R, Ferguson E, Ryan C, Williams JMG. Psychological processes and repeat suicidal behavior: A four-year prospective study. J Consult Clin Psychol. 2013;81(6):1137–1143. 10.1037/a0033751 23855989 PMC3933214

[52] Joiner Jr TE, Conwell Y, Fitzpatrick KK, et al. Four studies on how past and current suicidality relate even when “everything but the kitchen sink” is covaried. J. Abnorm. Psychol. 2005;114(2):291–303. 15869359 10.1037/0021-843X.114.2.291

[53] Newport D, Levey L, Pennell P, Ragan K, Stowe ZM. Suicidal ideation in pregnancy: Assessment and clinical implications. Arch Womens Ment Health. 2007;10:181–187. 10.1007/s00737-007-0192-x 17726640

[54] Suominen K, Isometsä E, Suokas J, Haukka J, Achte K, Lönnqvist J. Completed suicide after a suicide attempt: a 37-year follow-up study. Am. J. Psychiatry. 2004;161:562–563. 14992984 10.1176/appi.ajp.161.3.562

[55] Hawton K, Van Heeringen K. Suicide. Lancet. 2009;373(9672):1372–1381. 10.1016/S0140-6736(09)60372-X 19376453

[56] Black DW, Blum N, Pfohl B, Hale N. Suicidal behavior in borderline personality disorder: Prevalence, risk factors, prediction, and prevention. J Pers Disord. 2004;18(3):226–239. 10.1521/pedi.18.3.226.35445 15237043

[57] Cebrián I, Davia MA, Legazpe N, Moreno G. Mothers’ employment and child care choices across the European Union. Soc Sci Res. 2019;80:66–82. 10.2016/j.ssresearch.2019.02.003 30955562

[58] Morgenroth T, Hellman ME. Should I stay or should I go? Implications of maternity leave choice for perceptions of working mothers. J Exp Soc Psychol. 2017;72:53–56. 10.1016/j.jesp.2017.04.008

[59] Okimoto TG, Heilman ME. The ‘bad parent’ assumption: How gender stereotypes affect reactions to working mothers. J Soc Issues. 2012;68(4):704–724. 10.1111/j.1540-4560.2012.01772.x

[60] Alvesson M, Willmott H. Identity regulation as organizational control: Producing the appropriate individual. J Manag Stud. 2002;39(5):619–644. 10.1111/1467-6486.00305

[61] Paschetta E, Berrisford G, Coccia F, et al. Perinatal psychiatric disorders: An overview. AJOG. 2014;210(6):501–509. 10.1016/j.ajog.2013.10.009 24113256

[62] United Nations (UN). Transforming our world: The 2030 agenda for sustainable development. Resolution A/RES/70/1. Geneva: UN; September 2015.

